# Acid Denaturation Inducing Self-Assembly of Curcumin-Loaded Hemoglobin Nanoparticles

**DOI:** 10.3390/ma8125486

**Published:** 2015-12-11

**Authors:** Kaikai Wang, Juan Wang, Wenwen Hu, Yifan Zhang, Feng Zhi, Zaigang Zhou, Jinhui Wu, Yiqiao Hu

**Affiliations:** 1 State Key laboratory of Pharmaceutical Biotechnology & Medical School, Nanjing University, Nanjing 210093, China; kirk2008@126.com (K.W.); wangjuannm@163.com (J.W.); maso4704@163.com (W.H.); cyanii@yeah.net (Y.Z.); zhouzaigang1990@163.com (Z.Z.); 2 Modern Medical Research Center, Third Affiliated Hospital of Soochow University, Changzhou 213003, China; danielzhif@163.com

**Keywords:** drug carrier, hydrophobic forces, self-assemble, cell uptake

## Abstract

Hemoglobin is a promising drug carrier but lacks extensive investigation. The chemical conjugation of hemoglobin and drugs is costly and complex, so we have developed curcumin-loaded hemoglobin nanoparticles (CCM-Hb-NPs) via self-assembly for the first time. Using the acid-denaturing method, we avoid introducing denaturants and organic solvents. The nanoparticles are stable with uniform size. We have conducted a series of experiments to examine the interaction of hemoglobin and CCM, including hydrophobic characterization, SDS-PAGE. These experiments substantiate that this self-assembly process is mainly driven by hydrophobic forces. Our nanoparticles achieve much higher cell uptake efficiency and cytotoxicity than free CCM solution *in vitro*. The uptake inhibition experiments also demonstrate that our nanoparticles were incorporated via the classic clathrin-mediated endocytosis pathway. These results indicate that hemoglobin nanoparticles formed by self-assembly are a promising drug delivery system for cancer therapy.

## 1. Introduction

Being a major component of organisms, proteins have been the preferred biomaterials for drug delivery vehicles [1]. Firstly, proteins are known to undergo naturally controlled degradation processes. Moreover, they are relatively biocompatible and non-antigenic compared to synthetic polymers. Protein-based biomaterials have been widely investigated as drug delivery carriers, including gelatin, casein, albumin, and collagen [2,3]. For example, albumin is the most abundant plasma protein, exhibiting an average half-life of 19 days. Albumin-based nanoparticles can accumulate in tumor by both the “EPR” (enhanced permeability and retention) effect and the interaction with the gp60 receptor expressed on the surface of tumor cells [4,5]. Desai and co-workers developed a novel formulation of albumin-bound paclitaxel (nab-paclitaxel, Abraxane^®^; Celgene, NJ, USA) free of Cremophor EL^®^ (Aladdin, Shanghai, China). Abraxane^®^ was approved by US Food and Drug Administration (FDA) in 2005 for its dramatically enhanced water-solubility and maximum tolerated dose [6].

Hemoglobin (Hb) is roughly spherical, with a diameter of nearly 5.5 nm and a molecular weight of 64.5 kDa [7]. It is a tetrameric protein consisting of two pairs of α and β subunits, and each subunit contains a heme group in its hydrophobic region [8]. Hb is a traffic protein carrying oxygen and carbon dioxide *in vivo*. Hemoglobin has also been used widely as a blood substitute in clinical or preclinical research [9,10,11,12]. Therefore, Hb may have the potential to be a drug carrier for further application. There are several studies conjugating drugs into hemoglobin to form nanoparticles by chemical synthesis [13,14]. Complex synthesis steps and low drug loading are the main obstacles to the wide use of the chemical conjugation method. For example, Brookes, *et al.* had developed hemoglobin-ribavirin conjugates for targeting therapy purpose, but only six to eight ribavirin moieties attached per hemoglobin (less than 3% drug loading) [13].

To obtain protein-based drug-loaded nanoparticles, self-assembly of unfolded or partially unfolded proteins is convenient and cost-effective compared to chemical synthesis. Taking albumin as an example, Abraxane^®^ is prepared by emulsification through high-pressure homogenization followed by rotary evaporation, and the active agent paclitaxel (PTX) is dissolved in methylene chloride. This method is using homogenization to break up the intermolecular disulfide bonds between the sulfhydryl groups and make the resulting solution stable, with a particle size of 50–220 nm [6]. Another strategy is to break up the disulfide bond of albumin and expose the hydrophobic core to realize both the self-assembly of albumins and the encapsulation of the hydrophobic drug into hydrophobic center of the resulting nanoparticle. Gong *et al.* used β-mercaptoethanol (β-ME) and dithiothreitol (DTT) as denaturing reagents to successfully encapsulate paclitaxel and curcumin (CCM) into human serum albumin and demonstrated that the hydrophobic interaction was the main force of the assembly process of albumin nanoparticles [15,16,17]. Nevertheless, the use of denaturants and organic solvents largely limits the application of these assembly methods in the preparation of nanoparticles.

Hemoglobin undergoes structural changes at different pH values *in vitro*. It was reported that hemoglobin exhibited multistep unfolding transitions as pH declined [18,19]. Between pH 4 and 5.5, human hemoglobin dissociated partially, and it unfolded completely at pH < 2.5. De *et al.* demonstrated that the CD spectra of Hb at pH 1.9 showed a large departure from those at pH > 3 [20]. In this paper, we prepared curcumin-loaded hemoglobin nanoparticles (CCM-Hb-NPs) using the acid-denaturing method for the first time without adding toxic denaturants and organic solvents [21,22]. Additionally, the driving force was suggested to be hydrophobic interactions between hemoglobin and drugs. The uptake efficiency and cytotoxicity of CCM-Hb-NPs were significantly enhanced compared to free CCM solution. To clarify the enhanced effect of nanoparticles, the pathway of internalization was also characterized.

## 2. Results and Discussion

### 2.1. Hydrophobic Characterization of Hemoglobin under Acid Condition

**Figure 1:**
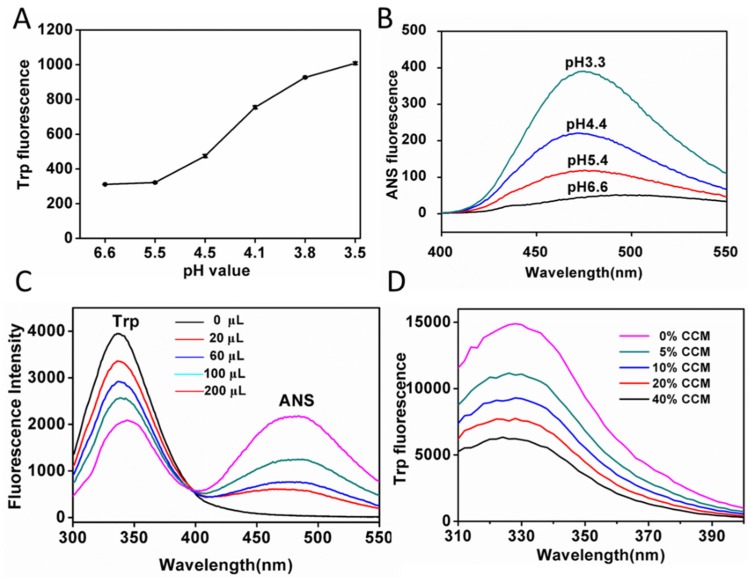
(**A**) Trp fluorescence changes of hemoglobin under different pH conditions (pH = 3.5, 3.8, 4.1, 4.4, 5.6, 6.6) (mean ± SD, *n* = 3). The excitation wavelength was 280 nm; (**B**) ANS fluorescence of hemoglobin under different pH conditions (pH = 6.6, 5.4, 4.4 and 3.3). The excitation wavelength was 380 nm; (**C**) Fluorescence Resonance Energy Transfer (FRET) from hemoglobin to ANS at pH 4.2. Fluorescence emission spectra of samples with hemoglobin (0.18 mg/mL) after adding 0, 20, 60, 100, 200 μL ANS (40 μM) scanned at room temperature. The excitation wavelength was 280 nm; (**D**) Trp fluorescence changes of hemoglobin after interacting with different amount of CCM (0, 5%, 10%, 20%, 40%, CCM:Hb, w/w) under pH 4.1.

The exposure of the hydrophobic area inside the hemoglobin was demonstrated by the Tryptophan (Trp) fluorescence and 1-(anilinon) aphthalene-8-sulfonic acid (ANS) [23,24]. Trp residues represented the hydrophobic domain of hemoglobin. Hemoglobin contains a number of buried tryptophan residues which are confined to hydrophobic areas close to the heme group [25]. Under physiological conditions, these residues are quenched from surrounding groups and have limited exposure to the solvent. Hence, weak fluorescence of Trp could be detected (shown in Figure 1A). As the pH descended, the fluorescence of Trp dramatically increased. This was most likely due to the exposure of buried Trp residues as the protein unfolded, indicating that low pH would expose the hydrophobic domain inside the protein.

Then ANS was used to observe the change in the exposed hydrophobic region of hemoglobin. ANS is minimally fluorescent in polar environments, such as aqueous solutions, but its fluorescence dramatically increased in nonpolar environments, including binding with the hydrophobic area of the protein [24]. Results showed that the fluorescence intensity of ANS increased when pH descended from 6.6 to 3.3 (Figure 1B and Figure S1). These results confirmed that acid-denatured hemoglobin had more hydrophobic moieties than native hemoglobin.

Once the tryptophan residue in the denatured hemoglobin is exposed to the solvent, the tryptophan fluorescence is quenched in the presence of ANS. The possible mechanism is the fluorescence resonance energy transfer (FRET) between the tryptophan residue and the ANS molecules due to overlap of the tryptophan emission and ANS excitation spectrum. As shown in Figure 1C, tryptophan fluorescence of hemoglobin was quenched more obviously during the addition of ANS. ANS can interact with hemoglobin both via hydrophobic and electrostatic interactions, depending on the pH studied. However, ANS is a positive-charged dye at the pH value of 4.2 and could not interact with hemoglobin by electrostatic interactions [26]. Therefore, the hydrophobic dye ANS could bind the hydrophobic region of proteins by hydrophobic interaction. So we have speculated that a hydrophobic drug CCM could also bind to the hydrophobic area of proteins by hydrophobic interaction. To substantiate that CCM bound to the hydrophobic area of hemoglobin to form CCM-Hb-NPs, we measured the fluorescence change of Trp during this process. As shown in Figure 1D, when interacting with more CCM, the fluorescence of Trp was quenched compared with free hemoglobin at pH 4.1. The quenching effect was probably due to interaction of CCM with hemoglobin, and the hydrophobic cavities of Hemoglobin might be the binding site of CCM. Taken together, these results indicate that hemoglobin could expose its hydrophobic domain under the acid condition and encapsulate hydrophobic molecular CCM to form nanoparticles.

### 2.2. Preparation and Characterization of CCM-Hb-NPs

**Figure 2:**
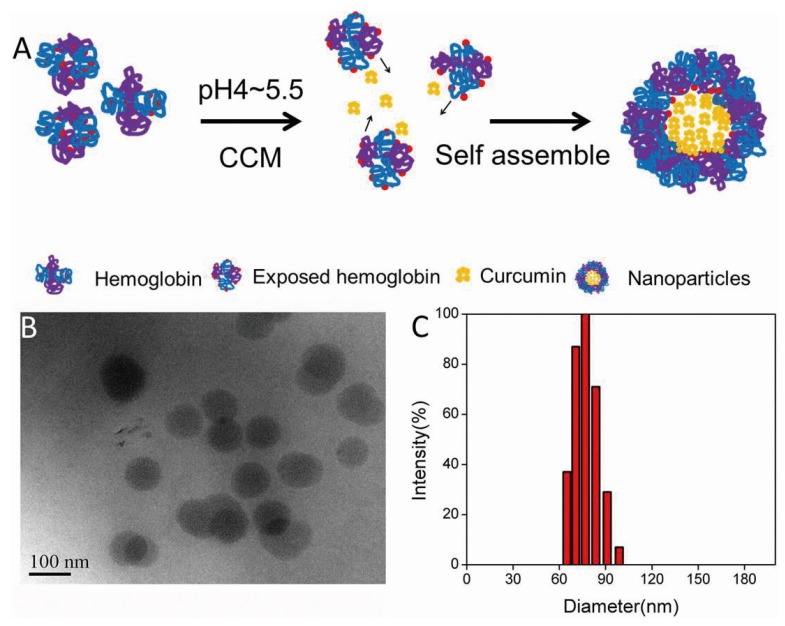
(**A**) Schematic illustration of preparation of CCM-Hb nanoparticles using the acid-denaturing method. Hemoglobin exposed its hydrophobic region under acid condition, and CCM interacted with exposed hemoglobin by hydrophobic force to form nanoparticles; (**B**) TEM photograph of CCM-Hb-NPs; (**C**) Particle size measured by dynamic light scattering (DLS), CCM-Hb-NPs were prepared at pH 4.0.

We prepared CCM-Hb-NPs using the acid-denaturing method. As shown in Figure 2A, the hydrophobic region of hemoglobin would be exposed at pH 4 to 5.5; therefore, the lipophilic drug could bind to the hydrophobic area and the nanoparticles were self-assembled. The morphology and particle size distribution of CCM-Hb-NPs (Figure 2B,C) were characterized by TEM and DLS, respectively. The TEM image showed that CCM-Hb-NPs were spherical with uniform particle diameter. The average diameter of CCM-Hb-NPs was around 89.0 nm, similar to the results obtained from TEM.

The major properties of CCM-Hb-NPs were summarized in Table 1 by varying drug feeding. The w/w ratio of CCM to hemoglobin was increased from 0% to 40% while particle sizes were also increased from 15.2 nm to 104.6 nm. Therefore, the particle size of CCM-Hb-NPs can be easily controlled by altering the amount of CCM. The drug loading of CCM-Hb-NPs could be up to 20.9%, which was much better than other chemical synthesis methods [13].

**Table 1:** Characteristics of CCM-Hb-NPs with changing drug feeding (pH 4.0).

CCM/Hb (w/w)	Particle Size (nm)	Zeta Potential (mV)	PDI	Drug Loading (%)	Encapsulation Efficiency (%)
0	15.2	+13.39	0.102	None	None
10%	74.7	+17.63	0.146	5.02	47.7
20%	82.5	+22.40	0.153	14.40	75.0
30%	92.5	+20.08	0.156	18.20	70.0
40%	104.6	+18.99	0.194	20.90	59.6

### 2.3. Effect of pH on the Particle Size and Stability of Nanoparticles

According to Table 2, the hemoglobin solution was adjusted to pH 6.6. After adding CCM, the solution exhibited a homogeneous-like state but precipitated soon (<0.1 h), indicating that hemoglobin nanoparticles could not be fabricated at this pH value. As the pH descended, the obtained CCM-Hb-NPs were more stable. Specifically, the particle sizes were 199.5 nm and 82.5 nm, and the stability times were 8 h and 168 h of nanoparticles obtained at pH of 5.6 and 4.1, respectively. When the pH was adjusted to 2.8, precipitations instead of hemoglobin nanoparticles emerged immediately. Hemoglobin would undergo structure changes under pH < 3 and failed to encapsulate CCM. CCM was a hydrophobic molecule and it remained insoluble at various pH values (Table S1 and Figure S2). De *et al.* demonstrated that the CD (circular dichroism) spectra of Hb at pH 1.9 showed a large departure from those at pH > 3 [20]. In addition, as the pH descended, the oxygen-binding affinity of Hb decreased. These results indicated that hemoglobin underwent a second-structure transition at pH < 3. Due to the structural change of hemoglobin by the UV spectrophotometer and CD spectra at different pH values, hemoglobin only slightly changes its properties at pH 4.1 (Figures S3 and S4). Therefore, we chose pH 4 to 5.5 to prepare CCM-Hb-NPs in case of inducing structural changes of hemoglobin. According to the results of the storage stability of nanoparticles, CCM-Hb-NPs were also very stable when prepared at pH 4.1 (Figure S5).

**Table 2:** Preparation of CCM-Hb-NPs at different pH values.

pH Value	Particle Size (nm)	Stability Time (h)
6.6	None	<0.1
5.6	199.5	8
4.1	82.5	>168
2.8	None	<0.1

### 2.4. CCM Extracted by Ethyl Acetate from CCM-Hb-NPs

**Figure 3:**
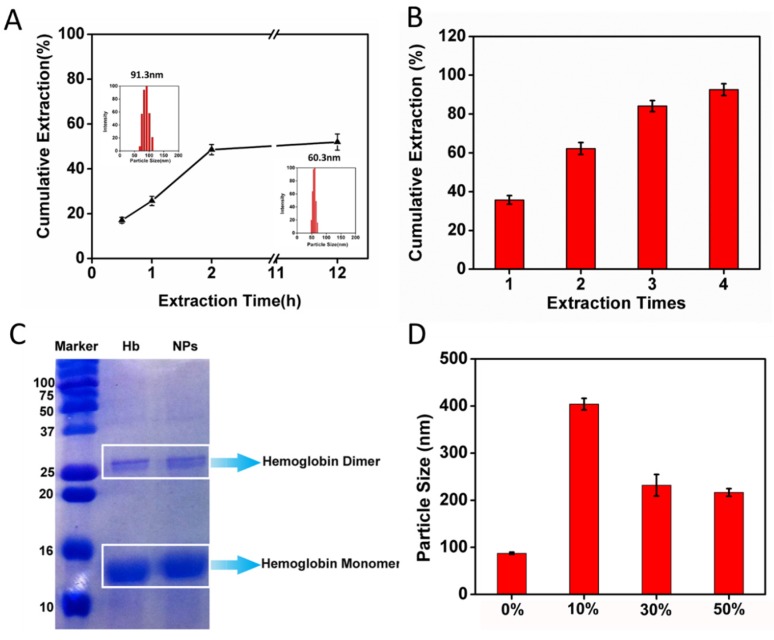
Further elucidation of the interaction forces that drive the formation of CCM-Hb-NPs. (**A**) Cumulative amount (%) of CCM extracted by ethyl acetate in 0.5 h, 1 h, 2 h and 12 h. Inset showed the particle size distribution of CCM-Hb-NPs solution mixed with ethyl acetate in 0 h and 12 h; (**B**) Cumulative amount (%) of CCM extracted by ethyl acetate in 1, which took 5 h for four times; (**C**) SDS-PAGE electrophoresis gels. Three lanes are of marker, native hemoglobin and NPs; (**D**) Particle size changes of nanoparticles mixed with 0%, 10%, 30%, 50% (v/v) of ethanol. (Mean ± SD, *n* = 3.)

To investigate the driving forces of the formation of nanoparticles, we used ethyl acetate to extract CCM from CCM-Hb-NPs based on the theory of similarity and intermiscibility. After adding CCM-Hb-NPs aqueous solution to ethyl acetate, there would be a demarcation line between the two layers (Figure S6). As shown in Figure 3A, after mixing with ethyl acetate, a portion of the CCM dissolved in the organic phase. The inserts were the particle sizes of the nanoparticle solution, which was 91.3 nm at the beginning and was reduced to 60.3 nm in 12 h, because the drug loading of CCM-Hb-NPs decreased. Then 51.90% CCM was extracted by ethyl acetate in 12 h, indicating that, as the concentration of CCM in ethyl acetate increased, the extraction capability of ethyl acetate weakened (Figure 3A). The extraction efficiency of curcumin by ethyl acetate was time-dependent. To extract CCM completely, fresh ethyl acetate was added every 1.5 h. As shown in Figure 3B, 92.57% of CCM were extracted after four times of extraction. The remaining solution after extraction of most of the CCM was similar to hemoglobin aqueous solution. For example, its particle size was 20 nm, as it was in the hemoglobin solution (Table 1). The results strongly suggested that CCM-Hb-NPs were formed through non-covalent forces, because ethyl acetate could not break up covalent bonds.

### 2.5. Size Change of CCM-Hb-NPs in Ethanol and SDS-PAGE of Nanoparticles

In order to confirm that non-covalent interactions stabilized our nanoparticles, we performed the SDS-PAGE of CCM-Hb-NPs and native hemoglobin. SDS could break hemoglobin into dimer and monomer form, but could not break up covalent bonds of proteins. So SDS-PAGE could be performed to substantiate the non-covalent cross-linking of hemoglobin during the nanoparticle formation process [27]. As shown in Figure 3C, native hemoglobin had two bands, monomer and dimer, as well as the nanoparticles. Nanoparticles are assembled by a large amount of hemoglobin via non-covalent forces, which would break into single hemoglobin in tetramer form under denatured conditions. There was no visible band of tetramer hemoglobin, because most tetramer-form hemoglobin broke into dimer form. However, not all of the dimers dissolved into monomers, so the band of dimer hemoglobin could be observed. Therefore, the results of the SDS-PAGE substantiated that the formation of nanoparticles was driven by non-covalent interactions.

To further identify the type of non-covalent interaction between proteins and CCM, we used ethanol to dissolve the nanoparticles. Ethanol could weaken the hydrophobic interaction [27]. When dissolved in 10%, 30% and 50% (v/v) ethanol, the particle size was 404.2 nm, 231.7 nm and 216.7 nm respectively (Figure 3D). At the low concentration of ethanol, the hydrophobic interaction between hemoglobin and CCM was weak, so the nanoparticles are not stable and CCM may leak out. With the increased concentration of ethanol, CCM dissolved again and the particle size of the CCM-Hb-NPs diminished. Results indicated that the incorporation of CCM into nanoparticles should be driven by hydrophobic forces.

### 2.6. Cell Uptake Study

**Figure 4:**
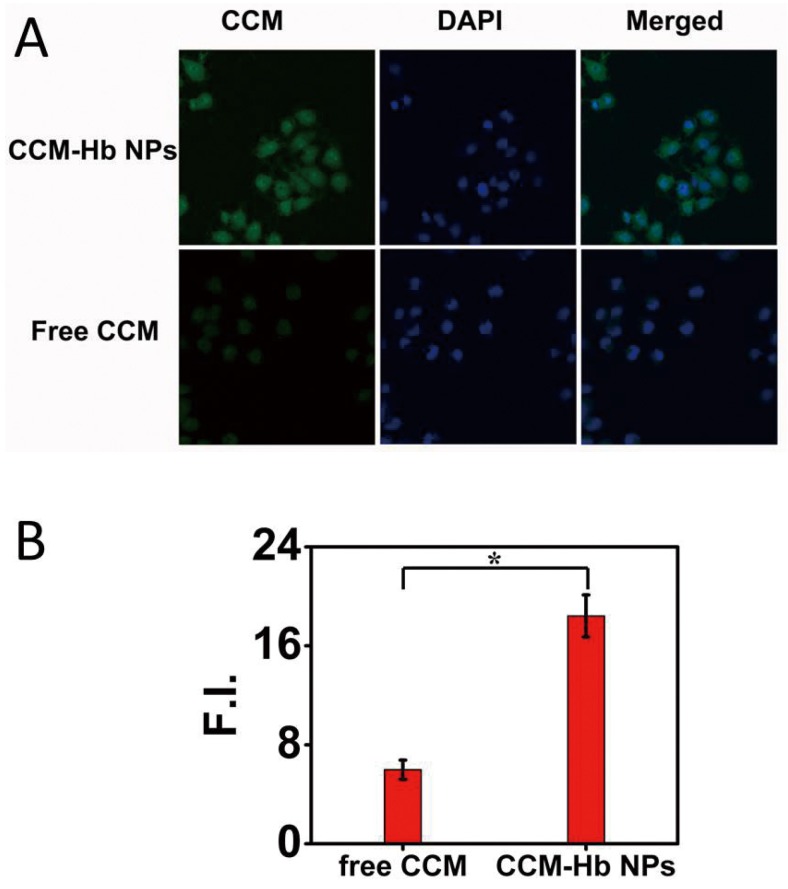
Uptake of CCM in MCF-7 cells. (**A**) Confocal fluorescence images of MCF-7 cells incubated with free CCM and CCM-Hb-NPs for 8 h, respectively; (**B**) Mean fluorescence intensity of CCM in each cell, calculated by Image J. (Mean ± SD, *n* = 30; * *p* < 0.05).

The cellular uptake of free curcumin and CCM-Hb-NPs by MCF-7 cells was observed by CLSM (confocal laser scanning microscope) after incubation for 8 h. In the CCM-Hb-NPs group, a larger amount of CCM was accumulated into the cytoplasm of the MCF-7 cells compared to the free CCM (Figure 4A). Results showed that the mean CCM fluorescence of each cell in the CCM-Hb-NPs group was significantly larger than in the free CCM group (18.42 ± 1.71 *vs.* 5.98 ± 0.98), indicating that better cell inhibition could be achieved in the CCM-Hb-NPs group than in the free CCM group (Figure 4B).

### 2.7. Cell Uptake Mechanism

**Figure 5:**
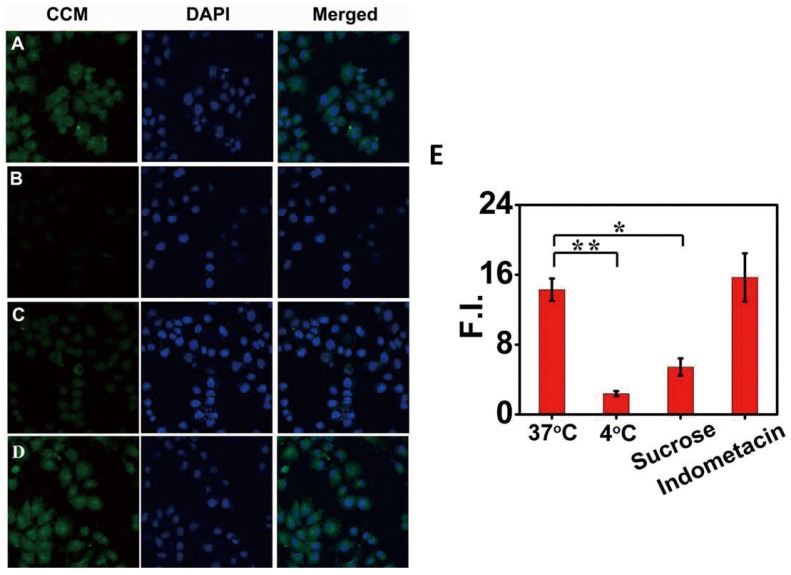
The uptake mechanism of CCM-Hb-NPs in MCF-7 cells: confocal fluorescence images of MCF-7 cells incubated with CCM-Hb-NPs at 37 °C (**A**); or 4 °C (**B**); or with sucrose (0.4 M) (**C**); or indomethacin (100 μM) (**D**); Mean fluorescence intensity of CCM in each cell calculated by Image J (**E**). (Mean ± SD, *n* = 30; * *p* < 0.05, ** *p* < 0.01).

To investigate the mechanism of enhanced cell uptake of CCM-Hb-NPs, we further investigated the effect of low temperature and endocytosis inhibitors including sucrose (a clathrin-mediated endocytosis inhibitor) and indomethacin (a caveolae-mediated endocytosis inhibitor) on cellular uptake efficiency [28]. The cellular uptake efficiency of CCM significantly decreased at 4 °C, manifested by the decreased fluorescence intensity of CCM compared to the control group (18.42 ± 1.71 *vs.* 2.38 ± 0.29). The results indicated that temperature played an important role in the cell uptake of CCM-Hb-NPs (Figure 5).

The cellular uptake efficiency of CCM at 37 °C was significantly inhibited by sucrose (a clathrin-mediated endocytosis inhibitor). In contrast, indomethacin, a caveolae-mediated endocytosis inhibitor, failed to inhibit the uptake of CCM. These results strongly indicated that our nanoparticles were internalized into the cytoplasm via the clathrin-mediated endocytosis pathway.

### 2.8. In Vitro Cytotoxicity Assay

**Figure 6:**
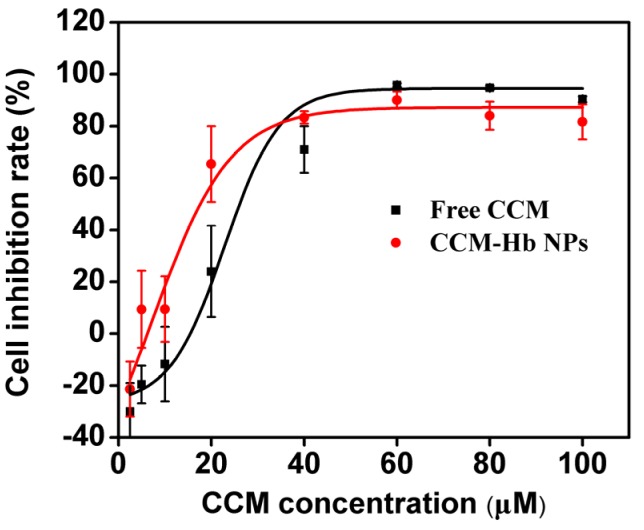
Cell viability of MCF-7 cells treated with different concentrations of free CCM or CCM-Hb-NPs and the cell inhibition rate curve was fitted using sigmoidal dose response fit by Origin 8.0 (mean ± SD, *n* = 6, * *p* < 0.05).

The cytotoxicity of free CCM and CCM-Hb-NPs was also investigated in MCF-7 cells (Figure 6 and Figure S7A). After incubation for 24 h, the cells were washed by PBS to remove CCM. CCM-Hb-NPs showed higher cytotoxicity (IC50 = 17.75 μM) compared with free CCM (IC50 = 26.13 μM), suggesting that CCM-Hb-NPs achieved higher activity. The reason could be the larger amount of CCM uptake for CCM-Hb-NPs than for free CCM.

Although CCM-Hb-NPs showed enhanced cytotoxicity compared with free CCM, hemoglobin had potential toxicity because of the heme part. Therefore, the cytotoxicity of hemoglobin alone was also conducted. Hemoglobin showed no toxicity on the MCF-7 cell line even at the concentration of 9 mg/mL at different pH values (Figure S7B). However, hemoglobin has potential renal toxicity *in vivo*, so further animal study should be conducted to evaluate the safety and side effects of CCM-Hb-NPs [29].

## 3. Materials and Methods

### 3.1. Materials

Human Hemoglobin (99%) was purchased from Sigma-Aldrich. Curcumin (CCM) (purity ≥ 99%) was purchased from Zelang Medical Technology Co., Ltd. (Nanjing, China) and cell counting kit-8 (CCK-8) was purchased from Dojindo (Kumamoto, Japan). The human breast cancer cell line (MCF-7) was purchased from Shanghai Institute of Cell Biology (Shanghai, China). Ethanol, ethyl acetate, 8-anilino-1-naphthalenesulfonic acid (ANS), salts, buffers and acids were analytic reagents and purchased from WanQing Chemical Classware Instrument Co., Ltd. (Nanjing, China).

### 3.2. Preparation of CCM-Hb-NPs

First, 80 mg of hemoglobin was dissolved in 20 mL water at room temperature. Then the pH of the protein solution was adjusted to 4.0 using 2 M HCl. Ten minutes later, CCM (7 mg/mL, ethanol as solvent) was slowly added to protein solution to prepare the nanoparticles. After that, the nanoparticle solution was ultrafiltered three times to remove free CCM and ethanol, and about 3 mL of final concentrated solution was obtained for further use.

Spectrophotometric method was used for the analysis of CCM amount in NPs. Briefly, the CCM-Hb-NPs were decomposed by acetate. After that, the mixed solution was diluted in 50-fold volume of chloroform and sonicated for 15 min to extract the CCM completely. The drug concentration was measured by reading the absorbance at λ = 419 nm using UV spectrophotometer (UV-2450, Shimadzu, Kyoto, Japan) and calculating from the standard curve of CCM in chloroform. The concentration of hemoglobin was detected by Coomassie brilliant blue.

The drug loading percentage (DL) and encapsulation efficiency (EE) were calculated according to:

(1) $$\text{DL}\%=\frac{\text{Weight of the drug in NPs}}{\text{Weight of the NPs}}\times 100\%$$

(2) $$\text{EE}\%=\frac{\text{Weight of the drug in NPs}}{\text{Weight of the feeding drug}}\times 100\%$$

### 3.3. Structure Change of Hemoglobin at Different pH Values

Acid unfolding of human hemoglobin was performed by incubating the protein at pH 2.5–6.6 for 0.5 h (all samples reached their maximum unfolding values within seconds or minutes). The structure change of hemoglobin was detected by UV-visible spectroscopy. The second-structure of hemoglobin before and after forming nanoparticles was determined by circular dichroism.

### 3.4. Size and Morphology of CCM-Hb-NPs

The average particle size and size distribution were determined by dynamic light scattering (DLS; 90Plus, Brookhaven Instrum. Corp, New York, NY, USA). The data were obtained from the average of triplicate measurements. The solution of CCM-Hb-NPs was placed on a 200-mesh copper grid coated with carbon. After deposition, the grid was air-dried and the morphology of nanoparticles was observed by transmission electron microscopy (TEM, JEM-2100, JEOL, Tokyo, Japan).

### 3.5. Fluorescence Studies of Hemoglobin before and after Interacting with CCM

The exposure of tryptophan residues was detected by monitoring the fluorescence intensity of tryptophan in hemoglobin using an RF-5301PC fluorescence spectrophotometer (Shimadzu, Kyoto, Japan) at 0.5 nm intervals. Tryptophan emits fluorescence at 300 to 400 nm, under excitation at 280 nm. The hydrophobic dye ANS was used as an indicator of exposure of hydrophobic clusters for its high affinity to hemoglobin. Then 40 μM ANS was added to hemoglobin at pH 3.3, 4.4, 5.4 and 6.6, respectively. The fluorescence change of ANS (40 μM) alone was also conducted at pH 4.1, 5.3, 6.1 and 7.4. The fluorescence at 400 to 550 nm was recorded after 15 min (when the value was stable) at 0.5 nm intervals, with 380 nm as the excitation wavelength.

Hemoglobin solutions (0.2 mg/mL) at pH value of 4.1 were incubated with different amounts of CCM (0, 5%, 10%, 20%, 40% of Hb, w/w), and the fluorescence of the nanoparticles and free hemoglobin of the same concentration were determined using a fluorescence microplate (Infinite 200, Tecan, Männedorf, Switzerland) The excitation wavelength was 280 nm, and the emission spectrum ranged from 310 nm to 400 nm.

### 3.6. Fluorescence Resonance Energy Transfer from Hemoglobin to ANS at Acidic pH Value

Fluorescence resonance energy transfer studies were carried out using fluorescence spectrophotometer F-7000 (HITACHI, Tokyo, Japan). Hemoglobin solution was adjusted to 4.2 by 2 M HCl. Protein samples containing different amounts of ANS (40 μM) were equilibrated for 20 min at room temperature before recording. The excitation wavelength was 280 nm, and the emission was recorded from 300 to 550 nm. Hemoglobin concentration was 0.18 mg/mL for experiments.

### 3.7. Determination of pH-Dependent Particle Size and Stability

During the nanoparticle preparation process, the stability time of solution is a major parameter to evaluate whether nanoparticles were well prepared. “Stability” here means that during the process of nanoparticles preparation, if hemoglobin could load CCM well, the solution would show homogeneous state and no aggregation could be observed. If nanoparticle size was larger than 1 μm, we think that nanoparticles had shown precipitation and were not stable. CCM-Hb-NPs were prepared over a pH range from 4 to 7. At four different pH values (2.8, 4.1, 5.6, 6.7), the particle size of nanoparticles was measured by dynamic light scattering (DLS; 90Plus, Brookhaven Instrum. Corp, New York, NY, USA). The precipitate time of nanoparticles was also recorded. The size change of CCM-Hb-NPs diluted in PBS (pH 7.4) in 48 h was also detected by DLS.

### 3.8. CCM Extracted by Ethyl Acetate in CCM-Hb-NPs

The 0.2 mL CCM-Hb-NPs solution was mixed with 3 mL ethyl acetate through lightly shaking, and the concentration of CCM in ethyl acetate was measured by UV spectrophotometer (UV-2450, Shimadzu, Kyoto, Japan) and calculated from the standard curve of CCM in ethyl acetate.

### 3.9. Size Change and Electrophoresis Gels of CCM-Hb-NPs

The particle size of nanoparticles added with four concentrations of ethanol (0%, 10%, 30%, 50%) was measured by dynamic light scattering (DLS; 90Plus).

Hemoglobin of CCM-Hb-NPs was separated on 12% SDS-PAGE gel. Approximately 10 μL of CCM-Hb-NPs and native hemoglobin were loaded per well. The gel was stained with Brilliant Blue R-250 staining solution (Bio-Rad, Hercules, CA, USA) at 37 °C for 1 h in a shaker at 100 rpm and later destained by destaining solution (Bio-Rad) for up to 3 h at 37 °C.

### 3.10. Cellular Uptake of CCM-Hb-NPs

The cellular uptake of CCM-Hb-NPs could be determined by detecting the fluorescence of CCM using Olympus FV1000 confocal laser scanning microscope (Olympus Corporation, Tokyo, Japan). Briefly, the MCF-7 cells were seeded in 12-well plates (5 × 10^4^ cells/well) and cultured overnight. Then the cells were exposed to 10 μg/mL of free curcumin or CCM-Hb-NPs (same dose of curcumin). After culturing for 8 h, the cells were washed twice with PBS and fixed with fresh 4% paraformaldehyde for 10 min. The nuclei of cells were then stained with DAPI for 15 min. Finally, the cells were imaged by confocal laser scanning microscopy (Olympus Corporation, Tokyo, Japan) in FITC (for CCM) and DAPI channel, respectively.

### 3.11. Cell Uptake Mechanism of CCM-Hb-NPs

Before being exposed to CCM-Hb-NPs at a CCM concentration of 10 μg/mL, the cells were treated with 0.4 M sucrose (inhibitor of clathrin-mediated uptake) or 100 μM indomethacin (inhibitor of caveolae-mediated uptake) or at 4 °C. Then the cells were exposed to 10 μg/mL free curcumin or CCM-Hb-NPs (same dose of curcumin). After further culturing for 8 h, the cells were washed twice with PBS and fixed with fresh 4% paraformaldehyde for 10 min. The nuclei of cells were then stained with DAPI for 15 min. The cells were imaged by confocal laser scanning microscopy in FITC channel (for CCM) or DAPI channel, respectively.

### 3.12. In Vitro Cytotoxicity

The cytotoxicity of CCM-Hb-NPs against human breast cancer cell line MCF-7 was measured by Counting Kit-8 (CCK-8) assay. CCK-8 was used to determine the living cells. Briefly, the cells were seeded in 96-well plates (5 × 10^3^ cells per well in 100 μL media) and cultured overnight. The culture medium was replaced with a fresh one containing equivalent doses of free CCM and CCM-Hb-NPs of 2.5 μM, 5 μM, 10 μM, 20 μM, 40 μM and 60 μM, respectively. After incubation for 24 h, the culture medium was removed and cells were washed with PBS. Then 10% (v/v) CCK-8 solution in fresh medium was added and cells were incubated at 37 °C for 2 h. The absorbance of the solutions was then measured at 450 nm by a microplate reader (Safire; TECAN, Männedorf, Switzerland). Cells cultured in PBS were the control group. Inhibition Rate = (1-A value of CCM well/A value of control well) × 100%. Multiple groups were compared using one-way analysis of variance (ANOVA) followed by the Tukey-Kramer test for *post hoc* comparisons. Statistical significance was set at *p* < 0.05.

## 4. Conclusions

In this study, we firstly proposed hemoglobin as a drug carrier to encapsulate the lipophilic drug CCM via the self-assembly method. In addition, through extensive investigation of the formation mechanism of CCM-Hb-NPs, we concluded that hemoglobin assembled into nanoparticles driven by hydrophobic interactions. Firstly, we found that the fluorescence intensity of Trp in hemoglobin would augment in acidic environment, which indicated that low pH would expose the hydrophobic domain inside the protein. To further examine its hydrophobic pocket, we used the hydrophobic probe ANS to mark the hydrophobic region and measured the fluorescence of ANS. Results showed that the hydrophobic region was exposed as we speculated, in accordance with the Trp fluorescence augment. Then we successfully incorporated the lipophilic CCM into hemoglobin in pH 4 to 5.5, and the binding region was suggested to be the hydrophobic area of hemoglobin. The fluorescence of Trp was quenched as soon as CCM was added. The following extraction experiment using ethyl acetate demonstrated that CCM-Hb-NPs were formed through non-covalent bonds. Ethanol, which could weaken hydrophobic interactions, was also used to interact with the nanoparticles. Results indicated that the incorporation of CCM into nanoparticles should be driven by hydrophobic forces. After demonstration of the formation mechanism, we also performed cell cytotoxicity and uptake experiments, and the results confirmed that both the uptake efficiency and cytotoxicity were significantly enhanced. The endocytosis-inhibiting experiment proved that CCM-Hb-NPs were internalized through a clathrin-mediated endocytosis. Because CCM inhibits proliferation of various tumor cells both *in vitro* and *in vivo*, it has a significant clinical prospect as an anti-cancer compound. Our CCM-Hb-NPs enhanced CCM solubility and achieved higher anti-tumor activity compared with free CCM. Hemoglobin may be a good carrier for CCM, as hemoglobin could target to CD163-overexpressed macrophages and monocytes. In further study, the anti-tumor efficacy will be examined *in vivo*.

## Supplementary Information

The following are available online at www.mdpi.com/1996-1944/8/12/5486/s1. Figure S1: ANS fluorescence intensity change at different pH values. Figure S2: The photographs of CCM-Hb-NPs solution and free CCM (the same amount of CCM as CCM-Hb-NPs) in PBS at different pH values. Figure S3: The absorbance of hemoglobin solution at different pH values by ultraviolet spectrophotometer. Figure S4: Circular dichroism (CD) analysis of native hemoglobin (pH 7.4) and CCM-Hb-NPs (pH 4.1). Figure S5: Storage stability of CCM-Hb NPs. Figure S6: The photographs of CCM-Hb-NPs solution mixed with ethyl acetate in 0 h and 12 h. Figure S7: *In vitro* cytotoxicity. Table S1: CCM solubility at different pH values (1 × PBS adjusted by 1 M HCl).
