# Supplementary material for: Acid Denaturation Inducing Self-Assembly of Curcumin-Loaded Hemoglobin Nanoparticles
Source: Materials (Basel). 2015 Dec 11;8(12):8701–13. doi: 10.3390/ma8125486 (PMC5458822; doi:10.3390/ma8125486)
Supplement: Supplementary file 1 [file materials-08-05486-s001.pdf]

Supplementary Materials: Acid Denaturation Inducing Self-Assembly of Curcumin-Loaded Hemoglobin Nanoparticles

Kaikai Wang, Juan Wang, Wenwen Hu, Yifan Zhang, Feng Zhi, Zaigang Zhou, Jinhui Wu and Yiqiao Hu

Table S1. CCM solubility at different pH values (1 × PBS adjusted by 1 M HCl).

Samples	CCM-Hb-NPs	pH 7.4	pH 6.08	pH 4.96	pH 4.0	pH 2.54
Concentration of CCM (μg/mL)	1666.7	0.22	0.41	0.37	0.27	0.27

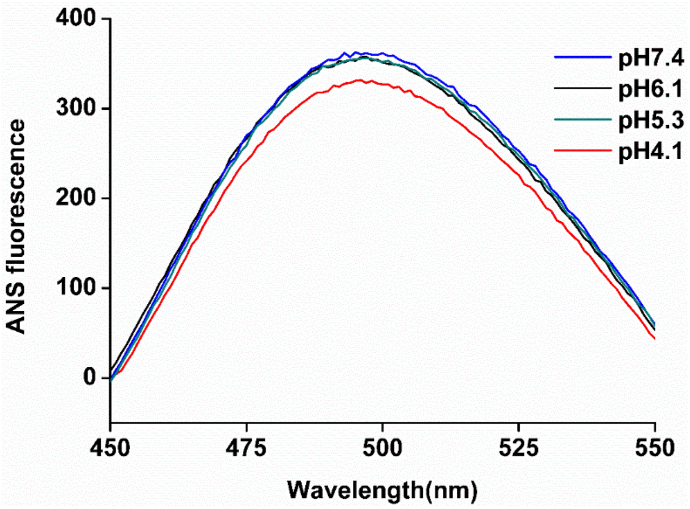

Figure S1. ANS fluorescences intensity change at different pH values.

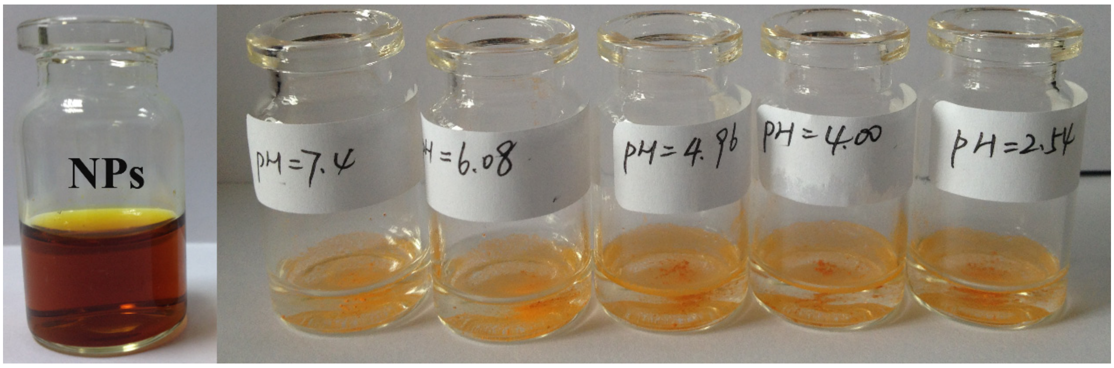

Figure S2. The photographs of CCM-Hb-NPs solution and free CCM (the same amount of CCM as CCM-Hb-NPs) in PBS at different pH values.

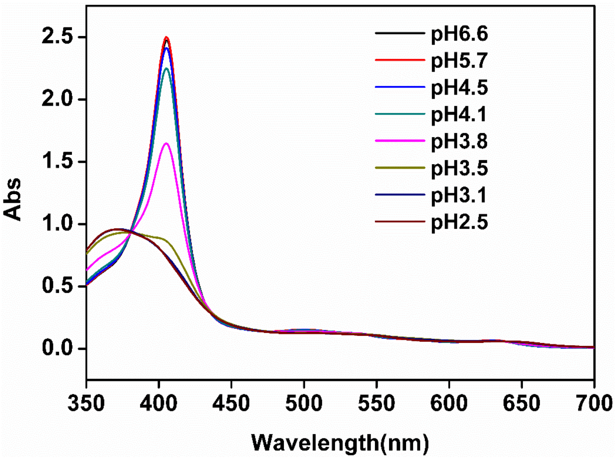

Figure S3. The absorbance of hemoglobin solution at different pH values by ultraviolet spectrophotometer.

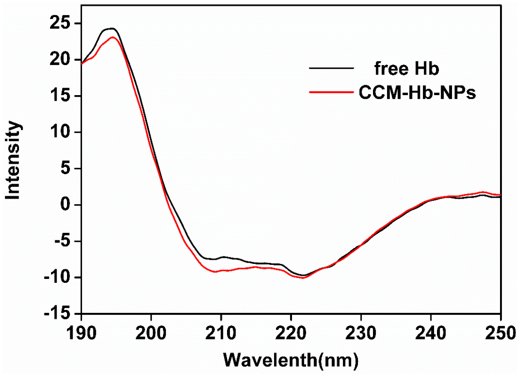

Figure S4. Circular dichroism (CD) analysis of native hemoglobin (pH 7.4) and CCM-Hb-NPs (pH 4.1).

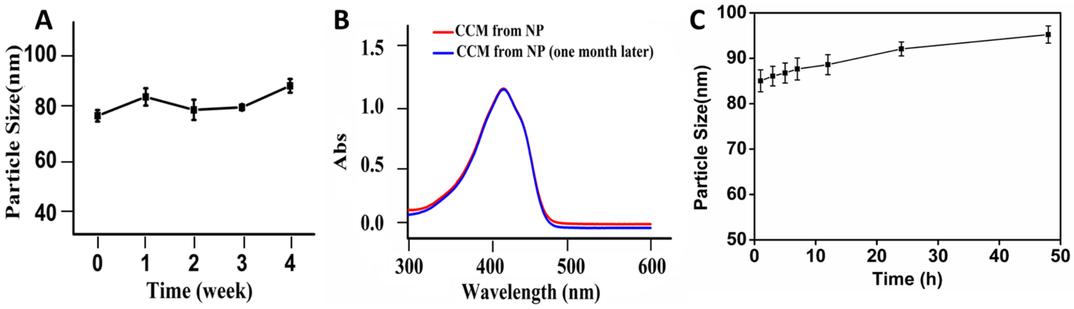

Figure S5. Storage stability of CCM-Hb-NPs. (**A**) Particle size change of CCM-Hb-NPs solution detected by DLS during one month; (**B**) UV spectrum of CCM extracted from CCM-Hb-NPs at 0 h and one month later; (**C**) Particle size change of CCM-Hb NPs diluted in PBS (pH 7.4) in 48 h.

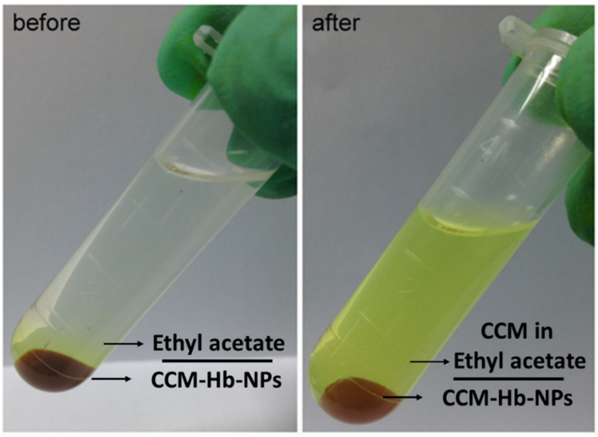

Figure S6. The photographs of CCM-Hb-NPs solution mixed with ethyl acetate in 0 h and 12 h

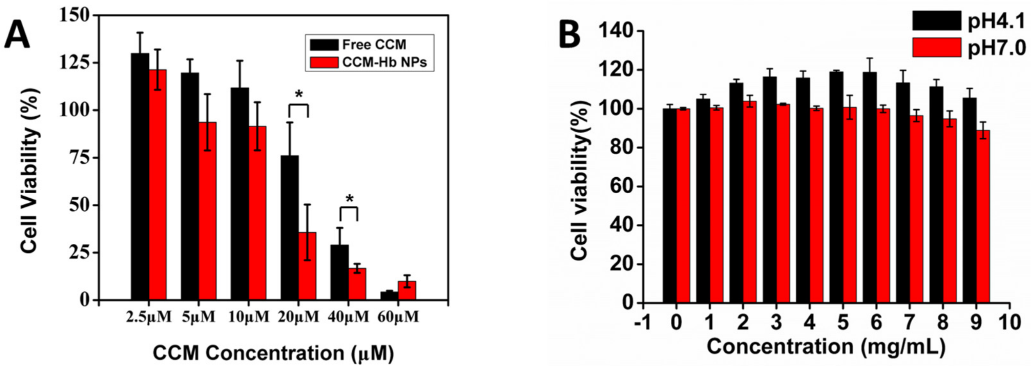

Figure S7. *In vitro* cytotoxicity. (**A**) Cell viability of MCF-7 cells treated with different concentrations of free CCM or CCM-Hb-NPs; (**B**) Effect of hemoglobin at different pH values on MCF-7 cells' viability measured by CCK-8 assay after 24 h incubation at 37 °C.
